# The Effect of in Ovo Injection Time and Dose of Maggot Oil from Black Soldier Fly (*Hermetia illucens*) on Hatching Rate, Growth Performance, and Biochemical Parameters of Broiler Chicks

**DOI:** 10.3390/ani15213115

**Published:** 2025-10-27

**Authors:** Yendouhamtchié Nadiedjoa, Xiaojuan Wang, Komi Attivi, Maxwell A. Okai, Qian Xin, Ahmed Mijiyawa, Clarice T. Maa Maa, Jingpeng Zhao, Hongchao Jiao, Komi Agboka, Hai Lin, Kokou Tona

**Affiliations:** 1 Key Laboratory of Efficient Utilization of Non-Grain Feed Resources (Co-Construction by Ministry and Province), Shandong Provincial Key Laboratory of Animal Nutrition and Efficient Feeding, Department of Animal Science, Shandong Agricultural University, Tai’an 271017, China; david.yendouhame@gmail.com (Y.N.); pmaxxies27@gmail.com (M.A.O.); 17863801275@163.com (Q.X.); ahmedmijiyawa4@gmail.com (A.M.); toukmaaclarice@gmail.com (C.T.M.M.); zjp@163.com (J.Z.); hongchao@sdau.edu.cn (H.J.); hailin@sdau.edu.cn (H.L.); 2 Laboratory of Poultry Production, Regional Center of Excellence on Avian Sciences (CERSA), University of Lomé, Lomé BP 1515, Togo; attivikomi1988@gmail.com (K.A.); kagboka@gmail.com (K.A.); jaktona@gmail.com (K.T.); 3 High School of Agronomy, University of Lomé (UL), Lomé BP 1515, Togo

**Keywords:** black soldier fly larvae oil, broiler chicks, hatchability, in ovo feeding, performance

## Abstract

**Simple Summary:**

Chicks expend a significant amount of energy during hatching, and energy deficits can lead to embryonic mortality. Vegetable oils are sometimes used for in ovo feeding as an energy source. In this study, in ovo feeding of oil from black soldier fly (BSF) larvae was carried out for Arbor Acres broiler embryos. The hatching rate and chick growth were improved. The in ovo feeding of hens’ embryos with BSF larvae oil also led to a decrease in LDL cholesterol levels. Therefore, the oil from BSF larvae can be used as an energy source for the embryonic nutrition of chickens.

**Abstract:**

There is an energy deficiency during the later stage of embryonic development, as the metabolic demands show an “explosive increase”. Vegetable oils are already used for in ovo feeding in poultry to provide energy for the embryos. What would be the effectiveness of animal oils used as alternative energy sources for the chicken embryo? To find out more, BSF larvae oil was used for in ovo feeding of the chicken embryo in this study. A total of 2300 Arbor Acres chicken eggs were used for incubation. On the tenth day of incubation, 2268 eggs were selected after candling and then divided into three groups for in ovo feeding in the yolk sac on the 11th, 14th, and 17th days of incubation. Each group was divided into seven lots, such as CON−, CON+, L0.1, L0.2, L0.3, L0.4, and L0.5. The CON− and CON+ were not injected. L0.1, L0.2, L0.3, L0.4, and L0.5 were pierced and then received the injection of 0.1 mL, 0.2 mL, 0.3 mL, 0.4 mL, and 0.5 mL of BSF maggot oil per egg, respectively. After hatching, 48 chicks from each lot of each group were housed in cages and then fed the same diet for six weeks. A better hatch rate and growth performance were observed for lots L0.1 and L0.2 compared to the other lots on the 14th and 17th days of incubation (*p* < 0.05). The injected lots showed reduced levels of low-density lipoprotein (LDL) cholesterol (*p* < 0.05). The injection of 0.1 mL BSF maggot oil on the 17th day of incubation had 0% embryonic mortality and 100% hatching success. In conclusion, BSF larvae oil can be used as an energy source for in ovo injection, with a dose of 0.1 mL on the 17th day of incubation being most effective and recommended.

## 1. Introduction

Nutrients are essential for the life and development of an animal. Generally, the chicken embryo obtains nutrients from the egg yolk, which mainly contains lipids [1]. Lipids are energy sources and are very important for chick hatching because the hatching period is a very energy-demanding stage [2,3]. The later stage of chicken embryo development is the fastest growing and most crucial period for functional maturity, with energy requirements increasing by three to five times compared to the initial and middle stages. After hatching, chicks are generally deprived of feed for a period of 24 to 84 h [4,5,6]. Numerous studies have reported that delayed access to feed reduces post-hatch performance in broilers [7,8,9,10,11]. Indeed, in ovo feeding is a technique that allows nutrients to be supplied to the chicken embryo during incubation. This helps the chick to overcome nutritional deficiencies after hatching. The study has found that in ovo feeding is an alternative method to increase the hatching rate and muscle growth weight of chicks [12]. Thus, Bhanja and Mandal [13] demonstrated in their study that in ovo injection influences embryonic development and the growth of chicks after hatching. The work of Kadam et al. [14] showed that in ovo injection of amino acids, carbohydrates, and vitamins improved hatchability and chick growth after hatching. Similarly, Ohta et al. [15] and Zielinska et al. [16] also showed that in ovo injection of amino acids supports embryonic development. According to Kpodo and Proszkowiec-Weglarz [17], in ovo feeding during early embryonic development directly influences organ formation, whereas during late development in ovo injection increases the energy reserves that will support the hatching process.

Vegetable oils have already been used to feed chicken embryos. Sulaiman and Tayeb [18] evaluated the effect of in ovo injection of 0.1 mL of some vegetable oils, such as rosemary oil, black cumin oil, olive oil, and almond oil, on the hatching rate and subsequent physiological responses of post-hatching broilers. Following the results of their work, hatching and post-hatching performance were improved in the test batches compared to the control lots. Currently, there has been a growing need to explore alternative energy sources, with insects gaining attention due to their favorable nutritional profile. Black soldier fly (BSF) maggots, which contain 40% to 45% protein and 17% to 49% lipids, are known as a promising feed ingredient in animal nutrition. However, to our knowledge, very few studies have been conducted on in ovo injections of maggot oil from BSF. Could in ovo injection of BSF larvae oil also improve hatchability and post-hatch performance of chicks? Further research is needed to find out. It is in this context that this work was undertaken to know the effect of in ovo oil injection of BSF maggots on the hatching rate and some physiological parameters after the hatching of broiler chicks.

## 2. Materials and Methods

The study was carried out in two experimental stages at the experimental farm of the College of Animal Science and Technology of Shandong Agricultural University, China.

### 2.1. Experiment 1 (Incubation)

For this study, a total of 2300 eggs from Arbor Acres broiler breeders of 50 weeks of age were obtained from a commercial farm (Weifang Hengrui Animal Co., Ltd., Weifang, China).

The birds were confined to ten hens per a cock in cages. Water and feed were provided ad libitum. All necessary vaccines and medications were provided according to the guidelines of the Veterinary Service. Before incubation, the eggs were numbered and weighed; the mean egg weight was 65.03 g. Incubation was performed in an incubator where the temperature and humidity were set to 37.8 °C and 60%, respectively, during the first 18 days of incubation. From the 19th to the 21st day, the eggs were subjected to the same temperature (37.8 °C), but the humidity was set to 70% in the hatchers. Indeed, on the 10th day of incubation, the infertile eggs were excluded, and 2268 fertile eggs were selected and then divided into three homogeneous groups, A, B, and C. Each group of 756 eggs was subdivided into seven lots of 108 eggs, labeled as CON−, CON+, L0.1, L0.2, L0.3, L0.4, and L0.5, for an in ovo injection of maggot oil from black soldier flies, i.e., six replicates of 18 eggs each. All the eggs were placed in the same incubator, and the eggs from each treatment of the different groups were distributed evenly across all the incubation trays. The in ovo injection of maggot oil from the black soldier flies was made through the yolk sac on the 11th, 14th, and 17th day of incubation for groups A, B, and C, respectively.

On the day of the injection, oils from BSF larvae were placed under the standard incubation temperature (37.8 °C) for 15 min before the injection. For this operation, the eggs were scanned with a light source, and the yolk sac was identified. The eggs were pierced using a 21-gauge needle drill 5 mm deep. The oil injection was performed with a semi-automatic syringe according to the method of Zhu et al. [19]. As soon as it was injected, the injection site of each egg was disinfected with 75% alcohol and then closed with paraffin. For each group, CON− had not been pierced or injected but was removed from the incubator and then replaced, along with the other batches. CON+ was punctured but had not received oil injection. On the other hand, each of the eggs in batches L0.1, L0.2, L0.3, L0.4, and L0.5 received 0.1 mL, 0.2 mL, 0.3 mL, 0.4 mL, and 0.5 mL of BSF maggot oil, respectively.

### 2.2. Oil from Black Soldier Fly Maggot

The maggot oil from the BSF was obtained from Sino Crown Biological Engineering Co., Ltd. (Qingdao, China) and stored at 4 °C for use. The fatty acid composition of BSF maggot oil is shown in Table 1.

### 2.3. Data Collection

From day 19 until hatching, external pipping data were recorded for each batch by observing the eggs every six hours. Early and late embryonic mortalities were determined after breaking the unhatched eggs and observing the developmental status of the embryos. The cumulative external pipping, hatch rates, and embryonic mortality rates of the chicks in each batch were calculated using Formulas (1), (2), and (3), respectively.

Cumulative External Pipping over time (%) = (total number of external pipping of egg on time)/Total number of incubated fertile eggs) × 100 (1)

Hatching (%) = (Number of hatched eggs/Total number of incubated fertile eggs) × 100 (2)

Total embryo mortality (%) = (Number of unhatched fertile eggs/Number of incubated fertile eggs) × 100(3)

### 2.4. Experiment 2 (Breeding)

After hatching, mixed-sex chicks (male and female) from each of the seven lots of each group were obtained according to the incubation setup. A total of 336 chicks per group, or 48 chicks by lot, were randomly selected due to six replications of eight, and placed in different cages measuring 95 cm × 75 cm × 60 cm. At the start, each lot had three cages housing sixteen chicks. During the growing phase of the chicks, the number of cages for each lot increased from three to six, housing eight chicks. The sanitation, temperature, lighting, and humidity were managed according to the requirements of broiler chickens. All were fed the same diet and had access to water ad libitum. The starter and grower feeds were purchased from Golden Rooster Hatching Co., Ltd. and Antimp Solar Feeds Co., Ltd. (Tai’an, China), respectively. The experimentation was carried out over six weeks.

### 2.5. Data Collection

The chicks were weighed at the beginning of the experiment and then weekly. The quantity of feed consumed was recorded by daily weighing of the feed distributed and the feed remaining unconsumed. The feed conversion ratio by weight was determined for each lot.

At the third and sixth weeks of age, six chickens were randomly selected from each lot due to one chicken per replication, and blood samples were taken by wing vein from chickens of each lot into dry tubes (without anticoagulants) and centrifuged at 3500 rpm for 10 min. The serum was obtained, and the biochemical parameters such as total protein (TP), glucose (GLU), albumin (ALB), alanine aminotransferase (ALT), aspartate aminotransferase (AST), triglyceride (TG), total cholesterol (TCHO), high-density lipoprotein (HDL), and low-density lipoprotein (LDL) were measured using the automatic biochemical analyzer (Hitachi High-Technologies Corp., Tokyo, Japan) with commercial reagent kits (Maccura Biotechnology Co., Ltd., Chengdu, China).

### 2.6. Statistical Analysis

The data were analyzed using GraphPad Prism version 8.02 (263) statistical software. One-way ANOVA was chosen for the data analysis of the effect of in ovo injection of BSF larvae oil on the chicks. Tukey’s test was chosen for comparisons between the different lots. The results were presented as means with standard error (Mean ± SEM). *p* < 0.05 was considered the threshold for significance.

## 3. Results

### 3.1. External Pipping

**Figure 1:**
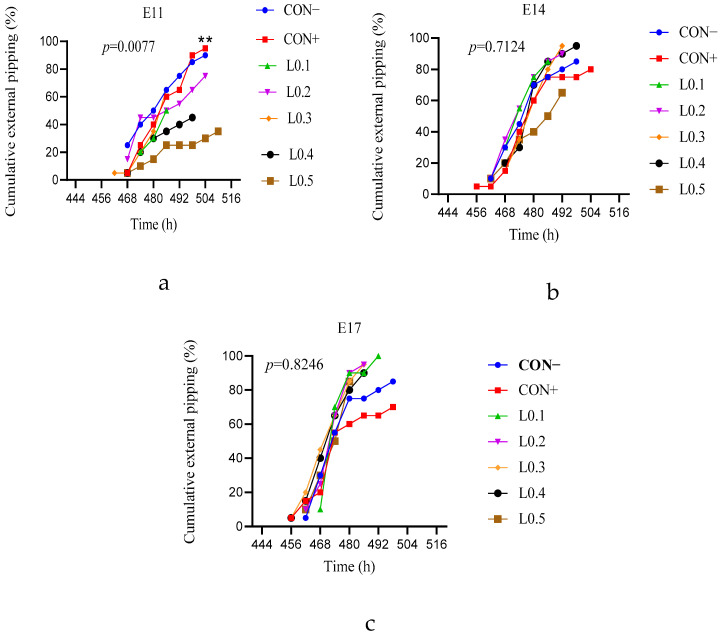
Cumulative rate of chicks’ external pipping from different lots of in ovo injection as a function of time. (**a**) 11th day in ovo injection; (**b**) 14th day in ovo injection; (**c**) 17th day in ovo injection. ** shows a significant difference (*p* ≤ 0.01). The results are represented as the mean value with pooled SEM (*n* = 6). CON−: Lot control without injection and without drilling; CON+: Lot control drilled but without injection; L0.1: Lot injected with 0.1 mL of BSF maggot oil; L0.2: Lot injected with 0.2 mL of BSF maggot oil; L0.3: Lot injected with 0.3 mL of BSF maggot oil; L0.4: Lot injected with 0.4 mL of BSF maggot oil; L0.5: Lot injected with 0.5 mL of BSF maggot oil.

Figure 1a shows the external pipping rate of the in ovo injection of maggot oil from black soldier flies on day 11 of incubation. The external pipping of lot L0.3 started first at the 462nd hour, but the other lots all started at the 468th hour. However, the control lots overtook all the test lots from the 486th hour to the 504th hour of incubation. In addition, the cumulative rate of external pipping of the control lots was statistically similar to that of L0.2 (*p* > 0.05), but significantly higher than those of L0.1, L0.3, L0.4, and L0.5 (*p* < 0.05).

Figure 1b shows the cumulative rate of external pipping of eggs after the in ovo injection of maggot oil from BSF on day 14 of incubation. The positive control lot was the first to start external pipping at the 456th hour and the last to end at the 504th hour of incubation, with a relatively lower cumulative rate than the other lots, except for the L0.5 lot. Even though at the 456th hour, the L0.1 and L0.2 lots overtook the curves of the other lots until the 486th hour of incubation, no significant difference was observed between all the lots (*p* > 0.05).

Figure 1c shows the cumulative rate of external pipping of eggs with the in ovo injection of maggot oil from BSF on day 17 of incubation. L0.1 was the last to start external pipping at the 468th hour, but it was above all the lots from the 474th hour until the 492nd hour of incubation. However, no significant differences were observed statistically between the different lots (*p* > 0.05). Overall, chicks hatched from eggs injected on incubation day 11 began external pipping 6 h later than chicks hatched from eggs injected on days 14 and 17.

### 3.2. Cumulative Hatching Rates

**Figure 2:**
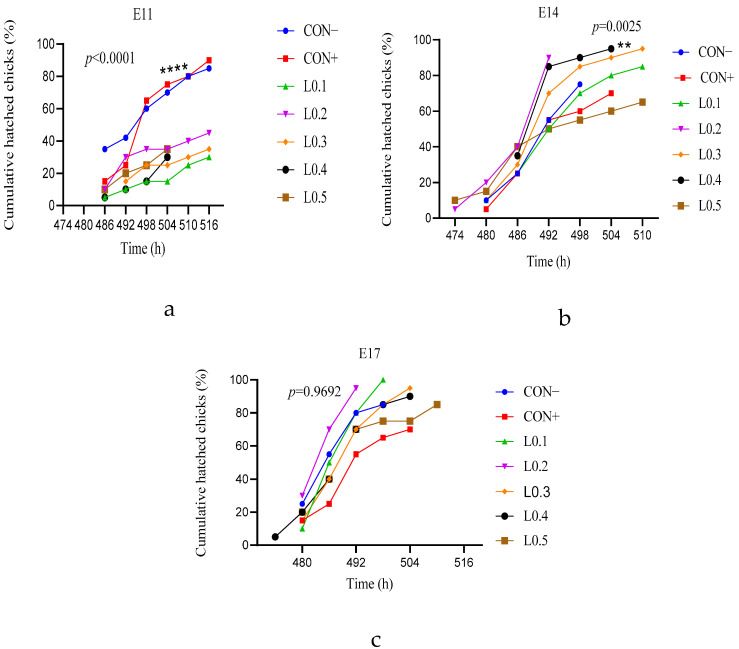
Cumulative hatched rate from different batches of in ovo injection as a function of time. (**a**) 11th day in ovo injection; (**b**) 14th day in ovo injection; (**c**) 17th day in ovo injection. ** shows a significant difference (*p* ≤ 0.01), **** shows a significant difference (*p* ≤ 0.0001). The results are represented as the mean value with pooled SEM (*n* = 6). CON−: Lot control without injection and without drilling; CON+: Lot control drilled but without injection; L0.1: Lot injected with 0.1 mL of BSF maggot oil; L0.2: Lot injected with 0.2 mL of BSF maggot oil; L0.3: Lot injected with 0.3 mL of BSF maggot oil; L0.4: Lot injected with 0.4 mL of BSF maggot oil; L0.5: Lot injected with 0.5 mL of BSF maggot oil.

Figure 2 shows the overall cumulative chick hatching rate of eggs with the in ovo injection of maggot oil from BSF. The cumulative hatching of chicks from the in ovo injection on day 11 of incubation is shown in Figure 2a. Hatching of the chicks from lot L0.3 started first at the 492nd hour of incubation, but hatching of the rest of the lots started from the 486th hour of incubation. Thus, except for L0.4, in which the last chick was hatched at the 504th hour, the hatching of the remainder of all other lots was completed at the 516th hour of incubation. In addition, the cumulative rates of the control lots were significantly better than those of all the test lots (*p* = 0.0001). The time between the emergence of the first and last chick in the control lots and the test lots was the same.

Figure 2b shows the cumulative hatching rate of the in ovo injection on day 14 of incubation. Hatching started first with L0.2 and L0.5 at the 474th incubation hour. Lots L0.3, CON−, and CON+ started hatching at the 480th hour, and later on, lot L0.4 started hatching at the 486th hour. Thus, from the 480th hour, the cumulative hatching rate of the L0.2 was better than the other lots until the 492nd hour, marking its end with a rate of 90%. But later, at the 510th hour, the hatching rate of L0.3 increased to 95%. However, the cumulative rate of batch L0.4 showed a significant difference compared to batch L0.5 and CON (*p* < 0.05). The remaining lots were similar to each other and to L0.4, L0.5, and CON (*p* > 0.05). Furthermore, the time between the hatching of the first and last chick in batch L0.2 was shorter (18 h) compared to the other lots.

Figure 2c shows the cumulative hatched chick rate from different lots that received in ovo injection on the 17th day of incubation. Except for L0.4, which was able to start hatching at the 474th incubation hour, all the other lots started at the 480th incubation hour. L0.2 performed better than all the other lots from the beginning until the 492nd incubation hour, when it ended with a cumulative rate of 95%. The time between the hatching of the first and last chick in batch L0.2 was shorter (12 h) compared to the other batches. But at the 498th hour, the hatching of L0.1 was better, with a cumulative rate of 100%. Then, at the 504th hour, the hatching of L0.3, CON−, L0.4, and CON+ terminated with cumulative rates of 95%, 90%, 90%, and 70%, respectively. Later at the 510th hour of incubation, the exit of the last chick from L0.5 was recorded, marking a cumulative rate of 85%. But no statistically significant differences were observed between the different lots (*p* > 0.05). Overall, the hatching process took 1.25 to 1.5 days for eggs injected on days 11 and 14, while it took 1 day for eggs injected on day 17.

### 3.3. General Chick Hatching Rate

**Figure 3:**
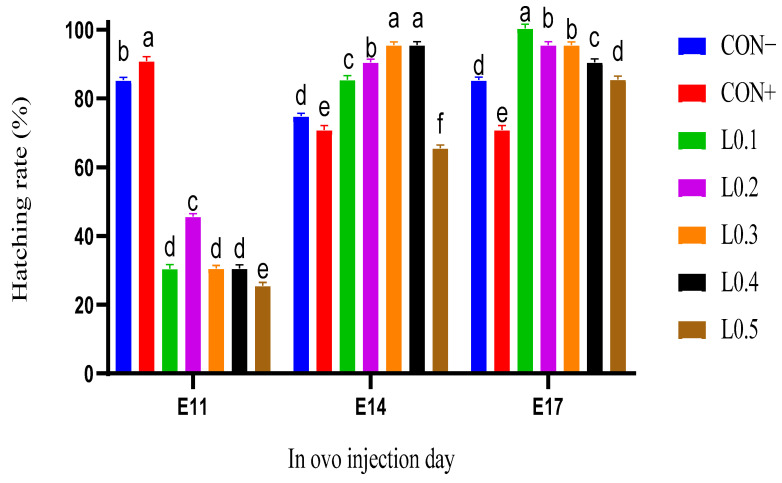
Hatching rate of chicks in different batches as a function of in ovo injection days. The results are represented as the mean value with pooled SEM (*n* = 6). Data with different letters indicate a significant difference (*p* < 0.05). CON−: Lot control without injection and without drilling; CON+: Lot control drilled but without injection; L0.1: Lot injected with 0.1 mL of BSF maggot oil; L0.2: Lot injected with 0.2 mL of BSF maggot oil; L0.3: Lot injected with 0.3 mL of BSF maggot oil; L0.4: Lot injected with 0.4 mL of BSF maggot oil; L0.5: Lot injected with 0.5 mL of BSF maggot oil.

Figure 3 shows the general hatching rate of chicks with the in ovo injection of maggot oil from BSF on the 11th, 14th, and 17th days of incubation. For the lots with the in ovo injection on day 11 of incubation, the positive control lot had a hatching rate of 90%, which was higher and significantly different from all the other lots, followed by the negative control lot (85%) (*p* < 0.05). In the test lots, the hatching rate of L0.2 was higher (*p* < 0.05) than L0.1, which was statistically similar to that of L0.3 and L0.4. The lowest hatching rate was recorded in lot L0.5 (25%).

At the 14th day of incubation, the hatching rates of the control lots were better than those of L0.5, which was the lot with the lowest rate (65%). On the other hand, all the other test lots had higher and significantly different hatching rates than the control lots (*p* < 0.05). Thus, L0.3 and L0.4 were the lots with the highest rate (95%).

On day 17 of incubation, the in ovo injection lot L0.5 had a hatching rate similar to that of the negative control lot (*p* > 0.05). But the other test lots had a higher and significantly different hatching rate than the control lots (*p* < 0.05). Thus, L0.1 had a better hatching rate (100%), followed by L0.2, which was similar to L0.3 but significantly different and higher than L0.4. The lowest hatching rate was recorded in the negative control lot with a value of 70%.

### 3.4. General Embryonic Mortality Rate

**Figure 4:**
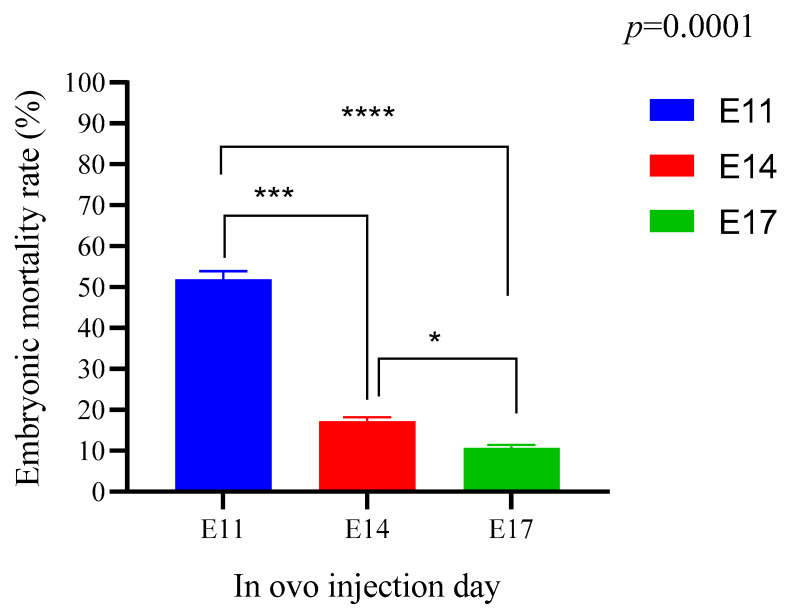
Embryonic mortality rate of the different batches according to the days of in ovo injection: (E11) 11th day in ovo injection; (E14) 14th day in ovo injection; (E14) 17th day in ovo injection. The results are represented as the mean value with pooled SEM (*n* = 6). **** shows a significant difference (*p* < 0.0001). *** shows a significant difference (*p* < 0.0002). * Shows a significant difference (*p* = 0.0342).

Figure 4 shows the embryonic mortality rate of the in ovo injection of maggot oil from black soldier flies on the 11th, 14th, and 17th days of incubation. The mortality rate was very high for the lots of the 11th day in ovo injection (52%), followed by the 14th day (17.6%) and finally on the 17th day (10.9%), with a significant difference (*p* < 0.05).

### 3.5. Early and Late Embryonic Mortality Rates

**Figure 5:**
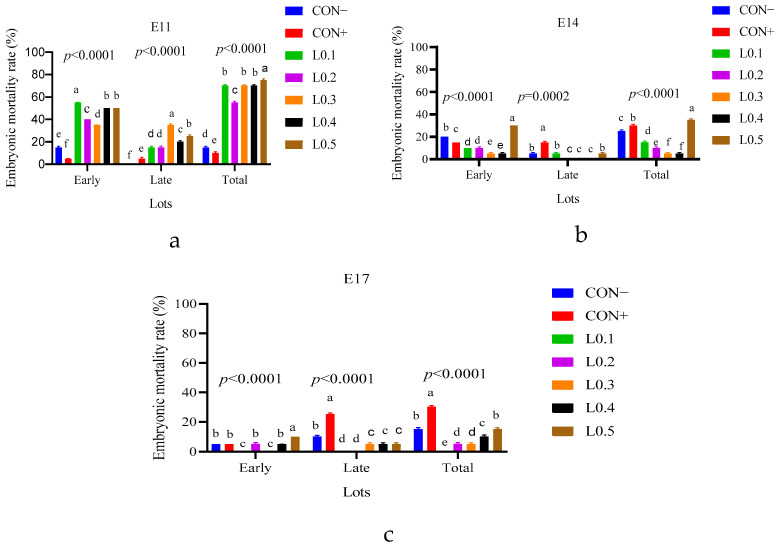
Early, late, and total embryonic mortality rates of batches from different days of in ovo injection: (**a**) 11th day in ovo injection; (**b**) 14th day in ovo injection; (**c**) 17th day in ovo injection. The results are represented as the mean value with pooled SEM (*n* = 6). Data with different letters indicate a significant difference (*p* < 0.05). CON−: Lot control without injection and without drilling; CON+: Lot control drilled but without injection; L0.1: Lot injected with 0.1 mL of BSF maggot oil; L0.2: Lot injected with 0.2 mL of BSF maggot oil; L0.3: Lot injected with 0.3 mL of BSF maggot oil; L0.4: Lot injected with 0.4 mL of BSF maggot oil; L0.5: Lot injected with 0.5 mL of BSF maggot oil.

Figure 5 shows the early, late, and total embryonic mortality rates of lots from different days of in ovo injection.

In the lots with in ovo injection on the 11th day (Figure 5a), there was a significant difference. Thus, the highest early mortality was observed in lot L0.1, followed by L0.4, which was similar to L0.5, then L0.2, L0.3, CON−, and CON+. Regarding late mortality, batch L0.3 had the highest rate, followed by L0.5 and L0.4, with a significant difference (*p* < 0.05). L0.1 had a late mortality rate similar to that of L0.2, which was also higher than the rate of CON+. Conversely, batch CON− recorded no late mortalities. Thus, the mortality rate was lower for the control batches compared to the test batches (*p* < 0.05).

Figure 5b shows the early and late embryonic mortality rates on the 14th day of incubation. Late mortalities were recorded in the CON−, L0.1, and L0.5 lots, which were similar to each other but significantly different than the CON+ lot. But early mortalities were recorded in all of the lots. Overall, lots L0.1, L0.2, L0.3, and L0.4 had lower embryonic mortality rates compared to the control lots, showing a significant difference (*p* < 0.0001). The embryonic mortality rate of batch L0.5 was higher than all the other lots. The lowest rates were observed in L0.3 and L0.4 lots, which were similar to each other.

Figure 5c compares early and late embryonic mortality rates of the in ovo injected lots on the 17th incubation day; no mortality was recorded in L0.1. The L0.2 lot also showed only early mortalities, while L0.3 showed only late embryonic mortalities. Early and late mortalities were recorded in CON−, CON+, L0.4, and L0.5, with the highest late mortality rate in the CON+ lot compared to all the others. Thus, L0.1 had no embryonic mortalities, and the rest of the tested lots showed lower rates of embryonic mortality compared to the control lots, with a significant difference (*p* < 0.0001).

### 3.6. Weight of Day-Old Chicks

**Figure 6:**
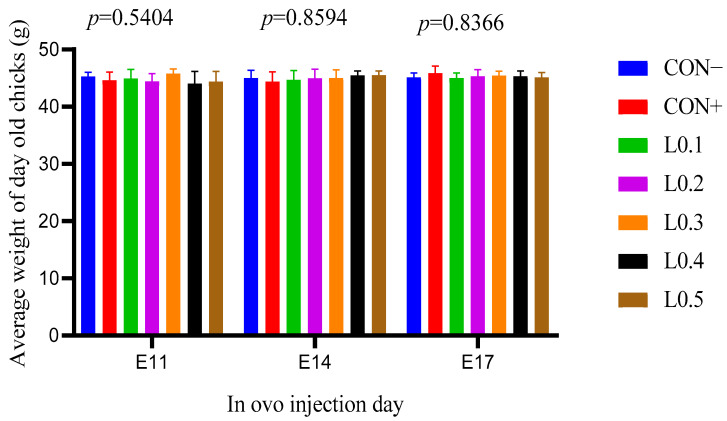
Average weight of day-old chicks in different batches as a function of in ovo injection days. The results are represented as the mean value with pooled SEM (*n* = 6). CON−: Lot control without injection and without drilling; CON+: Lot control drilled but without injection; L0.1: Lot injected with 0.1 mL of BSF maggot oil; L0.2: Lot injected with 0.2 mL of BSF maggot oil; L0.3: Lot injected with 0.3 mL of BSF maggot oil; L0.4: Lot injected with 0.4 mL of BSF maggot oil; L0.5: Lot injected with 0.5 mL of BSF maggot oil.

Figure 6 shows the average weight of day-old chicks with the in ovo injection of maggot oil from BSF. Whether it was the 11th, 14th, or 17th day of injection, no significant difference was observed between the different lots (*p* > 0.05).

### 3.7. Average Weekly Chick Weight

**Figure 7:**
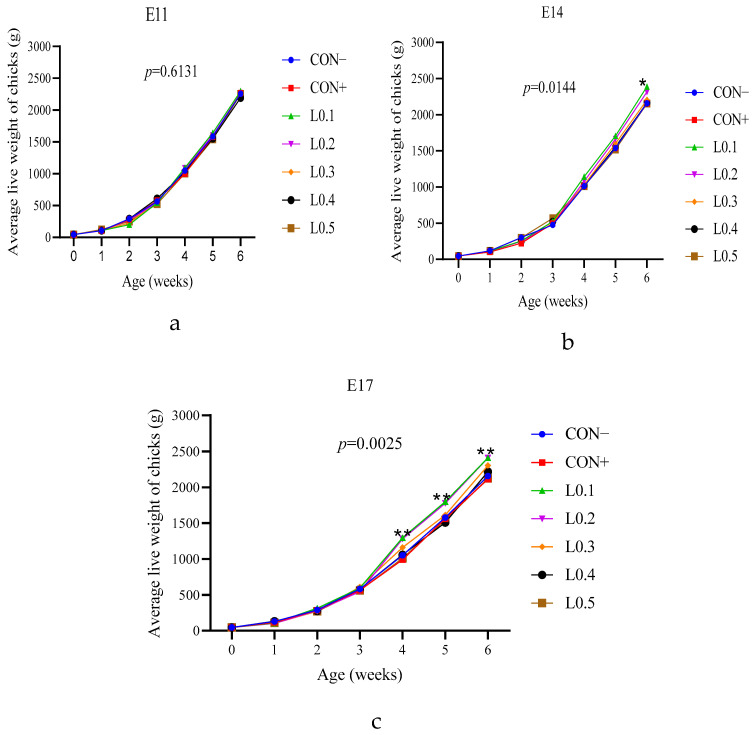
Average live weight of chicks as a function of age: (**a**) 11th day in ovo injection; (**b**) 14th day in ovo injection; (**c**) 17th day in ovo injection. The results are represented as the mean value with pooled SEM (*n* = 6). * Shows a significant difference (*p* ≤ 0.05), ** shows a significant difference (*p* ≤ 0.01). CON−: Lot control without injection and without drilling; CON+: Lot control drilled but without injection; L0.1: Lot injected with 0.1 mL of BSF maggot oil; L0.2: Lot injected with 0.2 mL of BSF maggot oil; L0.3: Lot injected with 0.3 mL of BSF maggot oil; L0.4: Lot injected with 0.4 mL of BSF maggot oil; L0.5: Lot injected with 0.5 mL of BSF maggot oil.

Figure 7 shows the average weekly chick weight of chicks with the in ovo injection of maggot oil from BSF. Figure 7a shows the weekly average chick weights after the in ovo injection on day 11 of incubation. From the first week to the sixth week of age, no significant difference in weight was observed between the chicks in the different lots (*p* > 0.05).

The average weekly weights of chicks after the in ovo injection on the 14th and 17th incubation days are shown in Figure 7b and Figure 7c, respectively. In both cases, the average chick weights of all lots were similar during the first three weeks of age. But, from the fourth to the sixth week of age, the weight of chicks in the lots L0.1 and L0.2, which were similar to each other, improved compared to the rest of the lots (*p* < 0.05). Overall, the final weight of chickens hatched from eggs injected on day 17 is greater than that of chickens hatched from eggs injected on days 11 and 14.

### 3.8. Feed Intake

**Figure 8:**
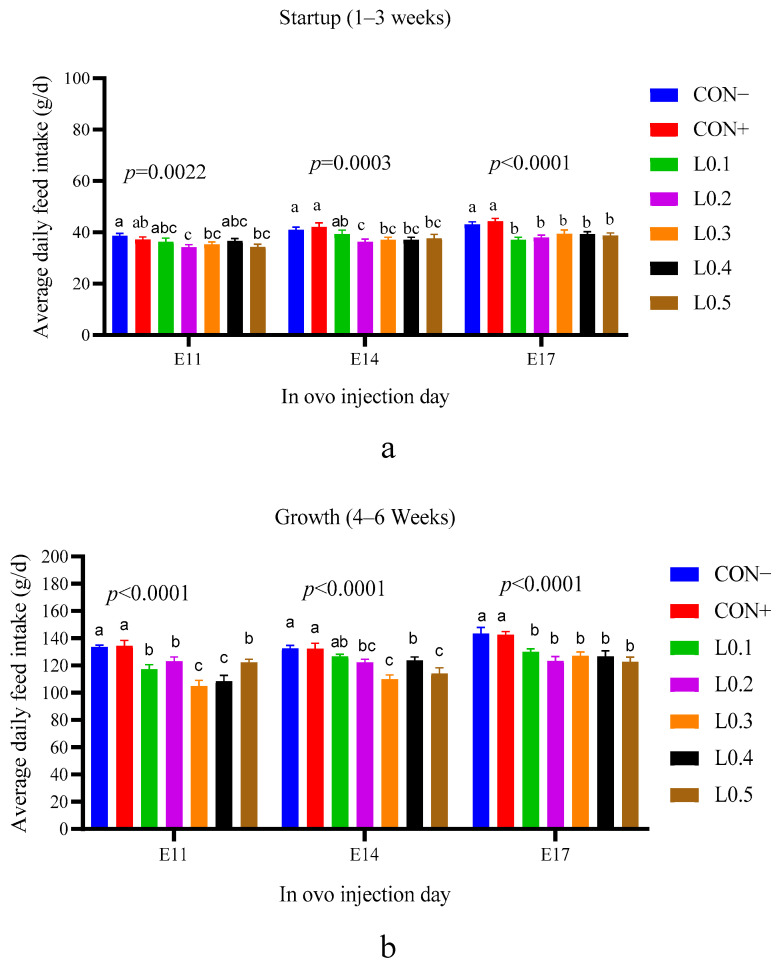
Average daily feed intake of chicks as a function of the different lots: (**a**) Startup phase, (**b**) Growth phase. The results are represented as the mean value with pooled SEM (*n* = 6). Data with different letters indicate a significant difference (*p* < 0.05). CON−: Lot control without injection and without drilling; CON+: Lot control drilled but without injection; L0.1: Lot injected with 0.1 mL of BSF maggot oil; L0.2: Lot injected with 0.2 mL of BSF maggot oil; L0.3: Lot injected with 0.3 mL of BSF maggot oil; L0.4: Lot injected with 0.4 mL of BSF maggot oil; L0.5: Lot injected with 0.5 mL of BSF maggot oil.

The feed intake during the start-up phase and growth of the chicks after the in ovo injection of maggot oil of BSF are presented in Figure 8a and Figure 8b, respectively.

In the in ovo injection group of the 11th day of incubation, the feed consumption of the CON+ lot was similar to the CON− lot and the other test lots, except the L0.2 lot. For the in ovo injection group of day 14 of incubation, the feed intake of the L0.1 lot was similar to the control lots and the remains of the other test lots, except for L0.2. The feed intakes of the in ovo injection control lots of day 17 of incubation were similar (*p* > 0.05) to each other but higher than those of all other test lots, which were also statistically similar. During the growth period (4–6 weeks), chicken feed consumption from groups with in ovo injection of the 11th, 14th, and 17th days of incubation was higher in the control lots compared to the test lots (*p* < 0.05).

### 3.9. Feed Conversion Ratio

**Figure 9:**
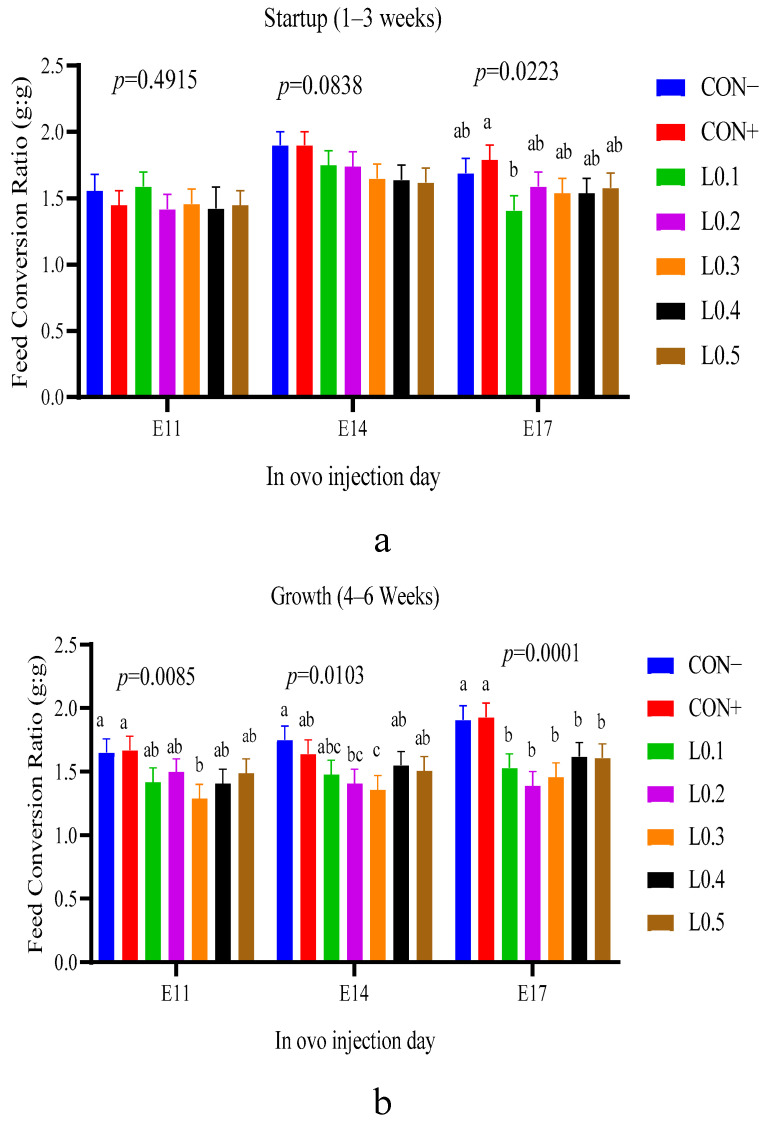
Feed conversion ratio of chicks as a function of the different lots: (**a**) Startup phase, (**b**) Growth phase. The results are represented as the mean value with pooled SEM (*n* = 6). Data with different letters indicate a significant difference (*p* < 0.05). CON−: Lot control without injection and without drilling; CON+: Lot control drilled but without injection; L0.1: Lot injected with 0.1 mL of BSF maggot oil; L0.2: Lot injected with 0.2 mL of BSF maggot oil; L0.3: Lot injected with 0.3 mL of BSF maggot oil; L0.4: Lot injected with 0.4 mL of BSF maggot oil; L0.5: Lot injected with 0.5 mL of BSF maggot oil.

The feed conversion ratio during the start-up phase (1–3 weeks) of chicks after the in ovo injection of black soldier maggot oil is shown in Figure 9a. No significant differences were observed between all lots resulting from the in ovo injection on incubation days 11 and 14 (*p* > 0.05). On the other hand, a significant difference was observed between some lots from in ovo injection on the 17th day of incubation (*p* < 0.05). The lowest feed conversion was given by lot L0.1, while the highest value was scored by lot CON+, but the rest of the lots were statistically similar (*p* > 0.05).

Figure 9b shows the feed conversion ratio of broiler chicks with the in ovo injection of maggot oil from BSF during the growth phase (4–6 weeks). For the 11th and 14th day in ovo injection lots, apart from the feed conversion of L0.3 which was different from the control lots, the rest of the test lots were statistically similar to the control lots. However, for the 17th day of incubation, the feed conversions of all test lots that were similar to each other were statistically lower than control lots (*p* < 0.05).

### 3.10. Blood Biochemical Parameters

Table 2 shows the blood biochemical parameters of chicks in the start-up phase for those with in ovo injection with BSF maggot oil on day 11 of incubation. No significant differences were observed between the different lots regarding total protein and albumin (*p* > 0.05). A significant difference was observed between some lots for the rest of the measured parameters. Thus, the ALT rate of the CON+ lot was similar to all other lots, but the CON− lot was different from the L0.1 and L0.4 lots (*p* < 0.05). AST in the L0.1 lot was higher than in all other lots (*p* < 0.05). The lowest rate was shown by L0.4, which was similar to L0.5. The glucose level of the L0.1 lot was lower and significantly different from the remainder of the lots (*p* < 0.05), which were also similar to each other. The triglyceride level was significantly higher in the L0.4 lot (*p* < 0.05), followed by CON− and L0.2 lots, then L0.1, L0.3, L0.5, and CON+. The lowest cholesterol level was recorded in the L0.1 lot. L0.2 and L0.5 were similar to each other but significantly different from the rest of the lots. The highest level was marked by L0.3, L0.4, and CON- lots (*p* < 0.05). No difference was observed between the control lots and the L0.2, L0.4, and L0.5 lots (*p* > 0.05). The control lots and test lots were similar for HDL (*p* > 0.05). The CON-, CON+, L0.2, and L0.3 lots had a similar LDL level that was also lower than the L0.4 lot.

An examination of the biochemical parameters of the start-up phase chicks of the 14th day in ovo injection with oil from black soldier maggot is shown in Table 3. There were significant differences between some lots for AST (*p* < 0.05). The highest level was recorded in lots L0.1 and L0.2, which were statistically similar to each other, followed by L0.3 and L0.5, which were also similar, and then L0.4. The lowest value was given by the lot controls. But no significant differences were observed between all the lots for the rest of the parameters (*p* > 0.05). Thus, the blood parameters were balanced.

Table 4 presents the results of the blood biochemical parameters of the start-up phase chicks that received an in ovo injection of maggot oil from BSF on the 17th day of incubation. AST and HDL showed a significant difference between different lots (*p* < 0.05). The AST value of L0.5 was higher compared to the other lots, followed by CON−, L0.1, and L0.4, which were statistically similar to each other, followed by L0.2, L0.3, and finally CON+. Concerning HDL, the highest value was recorded in the L0.1 lot but was statistically similar to all test lots and different from the control lots, which were also similar to each other. However, no significant differences were observed between all the lots for the rest of the parameters measured (*p* > 0.05).

Table 5, Table 6 and Table 7 show the results concerning the biochemical parameters of chicks that received the in ovo injection of maggot oil of BSF, respectively, on the 11th, 14th, and 17th days of incubation, during the grow period. Whether it was injected on the 11th, 14th, or 17th day of incubation, a significant difference was marked only for LDL cholesterol, with a relatively high level for the control lots compared to the injected lots (*p* < 0.05). But no significant differences were observed for the rest of the parameters measured in different lots (*p* > 0.05).

## 4. Discussion

Hatching is a phenomenon in which the chicken embryo goes through several processes, namely assuming the right position, internal pipping, and external pipping, to be able to come out of the eggshell and lead a free life. All these physical steps require enough energy. The cumulative external pipping scores of the control lots from the 11th day of incubation in ovo injection were better than the test lots. Aberbour et al. [20] had suggested that an in ovo injection of three microliters of rosemary essential oil into quail eggs on the 9th day of incubation was toxic to the embryo and led to a decrease in hatchability. Compared with the different doses of BSF larvae oil used in the present study, this suggests that the various doses have negatively affected the embryos of injections on the 11th day of incubation. But as age increases, the embryo can tolerate certain doses. This is why the external pipping of lots L0.1, L0.2, and L0.3 from the in ovo injection on the 14th day of incubation was better compared to the control batch. Similarly, except for lot L0.5, all external pipping of all test batches from the in ovo injection on the 17th day of incubation was also better. According to Kpodo and Proszkowiec-Weglarz [17], in ovo feeding during late embryonic development increases the energy reserves and supports the hatching process. With the dose being appropriate at this stage, BSF larvae oil provided the necessary energy, which allowed chicks from the test lots to break the eggshell. The external pipping results corroborate the hatchability results.

The group injected on the 11th day of incubation gave a better hatching rate in the control lots compared to all the test lots. On the other hand, the in ovo injected groups of the 14th and 17th days of incubation have the best hatching rate for the lots injected with the doses of 0.1 mL, 0.2 mL, 0.3 mL, or 0.4 mL. The cumulative rate of chicks in the L0.2 and L0.1 lots which were injected on the 14th and 17th day of incubation was higher than that of the other lots, and the time between the emergence of the first and last chick was also shorter compared to the different lots, indicating a reduction in the hatching window, which is important because this allows for obtaining high-quality chicks and the best post-hatching performance. However, the highest hatching rates were recorded for the lots injected on the 17th day of incubation, where a 0.1 mL dose was considered a success index, yielding a hatching rate of 100%. Indeed, lipid nutrients are an energy source. By injecting oil from BSF maggots in ovo, additional energy is produced that can be used in the chicken embryo for the best hatching. This was the case for the in ovo injected groups on days 14 and 17 of incubation. In ovo feeding lots, the hatching rate of those injected on the 17th day was better than those of the 14th day, which would mean that the amount of energy produced by the oil of BSF maggots was sufficient to support the 17th day embryo until hatching, more so than that of the 14th day embryo. Similarly, Sulaiman and Tayeb [18] injected 0.1 mL of vegetable oils in ovo on the 18th day of incubation, and also observed an improvement in the hatching rate. However, the difference in hatching rates between treatments shows that the injection dose has an effect on the chicken embryo, and this was negative for high doses, particularly the volume of 0.5 mL, showing a low rate for each group. Regardless of the type of substance used, an appropriate dose is required for in ovo injection. Other studies have already suggested this with substances other than maggot oil. When Zhai et al. [21] injected 0.1, 0.4, 0.7, or 1.0 mL of diluent carbohydrate into eggs at 18.5 days of incubation, the hatching rate decreased according to the dose, and they suggested that to achieve a 90% hatch rate, the injection volume should not exceed 0.4 mL for fructose or sucrose and should not exceed 0.7 mL for glucose, maltose, or dextrin.

The results of hatching rates are related to the results of embryonic mortality rates shown in Figure 4. The mortality rate was low for all lots injected on the 17th day of incubation. During the hatching period, the embryo needs energy to break the eggshell. A deficit of energy makes this operation difficult, even leading to late mortality. This explains the increase in the late mortality rate of the control lots compared to the test lots injected on the 17th day of incubation (Figure 5c).

At hatching, no significant difference was observed for chick weights in all lots. Thus, the maggot oil from BSF did not play a significant role in the process of muscle development. Similar results were reported by many works. Ma et al. [22] did not find a significant difference in day-old chick weight when they injected flaxseed oil and soybean oil in ovo. Also, Samson et al. [23] did not find a significant difference in the weights of one-day-old chicks when they performed the in ovo injection of 0.2 mL of essential oil into the amniotic fluid of broiler chicken embryos on the 18th day of incubation. Some substances other than oil used for in ovo injection did not result in a difference in the weight of chicks at hatching. This is the case for an in ovo injection of L-glutamine into the amniotic sac of embryos performed on the 18th day of incubation [24]; in ovo injection of gamma-aminobutyric acid performed in the amniotic fluid of the breeding chickens’ eggs at 17.5 days of incubation [25]; and in ovo injection of creatine monohydrate carried out in the air chamber of broiler chicken eggs at 17.5 days of incubation [26]. However, when Majida et al. [27] performed an in ovo injection of omega-3 oil at different doses, such as 0.1 mL, 0.2 mL, and 0.3 mL per egg, into the amniotic fluid of broiler chicken eggs at 18 days of incubation, the weights of the day-old chicks in the injected groups were higher compared to the control. The difference in this result compared to the result of this current work may be due partly to the difference in injection time (18th/17th day) or to the difference in the fatty acids.

The chick weights of all the 11th day lots of injection were similar. On the other hand, the injected lots L0.1 and L0.2 had better weights compared to the other lots on the 14th and 17th day of injection. This showed the positive long-term impact of in ovo injection of BSF maggot oil in chick production. Sulaiman and Tayeb [18] also reported similar results when they injected natural oils. In addition, due to the antimicrobial role of lauric acid in BSF maggot oil, it can also act on the intestinal ecosystem by reducing the number of pathogenic microorganisms in the gastrointestinal tract of chickens. The stability of the intestinal microflora promotes the ideal pH for the activation of digestive enzymes [28].

Feed consumption influences the growth and weight of an animal. During the start-up phase, on the 11th day of incubation, feed intake of all lots was practically similar. The difference was remarkable between the control lots and the test lots of in ovo injection on the 14th and 17th days of incubation. On the 11th day of incubation, the yolk sac of the hen’s egg is still filled with substance, so injecting another substance is only a substitution and not an addition. But as time passes, a certain amount of yolk sac nutrient will be used for physiological needs, and the remaining space can be used for in ovo nutrient addition. After hatching, the chick has nutrients in the yolk sac, which are adapted to the digestion process and can be used for the first 5 days of its life [29]. In addition, the first days of a chick’s life are also a time of dietary transition, and chicks take time to adapt to solid feed. For this reason, on the 11th day of incubation, the feed intakes of all the chicks of the in ovo injected lots were similar. On the other hand, the decrease in feed intake of chicks in the test lots compared to the control lots on the 14th and 17th day of incubation is explained by the fact that, at this time, in ovo injection allows nutrients to be added quantitatively or qualitatively that can accompany the chick for a long time after hatching, therefore, the chicks’ feed intake decreased. However, even if the difference in feed intake was observed between the injected lots of the chicks of the 17 day incubation, it should be noted that no significant difference in average body weight was noticed between all the chicks of all lots of all injection times. During the hatching of domestic pigeons, Zhu et al. [30] evaluated the effect of in ovo feeding L-lysine and also found no significant difference between the lots.

For all in ovo injection timings, the injected groups consumed less feed at the growth stage compared to the control groups. The decrease in feed intake in the treated groups suggests that the oil from BSF larvae may have developed the intestinal cells. In ovo injection allows for directly providing chicks with essential nutrients to optimize the growth and maturation of the digestive tract. Chen et al. [31] had already found that chicks receiving in ovo methionine injection into the yolk sac showed better intestinal development and growth performance. Indeed, intestinal cells regulate nutrient absorption through the intestinal wall. Furthermore, the enteroendocrine cells of the intestine produce hormones such as serotonin, somatostatin, and cholecystokinin, which regulate appetite and metabolism, thus influencing feed consumption. Thus, the work of El-Sayed et al. [32] has shown that the use of certain essential oils in the chick ration leads to the development of intestinal cells.

The feed conversion ratio was also lower for all test lots compared to control lots, particularly for the chicks in the growth phase. This allows us to affirm that the in ovo injection of maggot oil from BSF has led to the development of intestinal cells, resulting in the valorization of nutrients and a decrease in the feed consumption. These results are consistent with the work of Kim et al. [33], who also showed that the use of oil from BSF larvae as an alternative fat source in broiler feed improves the feed conversion ratio. Ma et al. [22] also found that feeding soybean oil and flaxseed oil in ovo improved feed conversion in broilers. In addition, when El-Sayed et al. [32] evaluated the stimulating effect of Bacillus subtilis essential oil in broilers, they also found that the oil improved feed conversion.

Blood serum parameters are indicators that make it possible to assess the state of health of animals. The activities of AST and ALT are often biomarkers of liver health [34]. In the growth phase, whether it is the in ovo injection on the 11th, 14th, or 17th day of incubation, no significant differences were observed between the test and control lots for AST and ALT. Thus, the in ovo injection of maggot oil from BSF had no negative impact on the liver, which is a main organ for the digestion of lipids. Although the AST concentration showed a significant difference during the start-up phase, it should be noted that AST can be secreted by several organs, such as muscles, kidneys, and the brain, not just the liver. Therefore, liver dysfunction cannot be determined only based on the results of AST. Total protein and albumin also did not show a significant difference in all lots during both the start-up and growth phase. Serum protein and albumin levels are endpoints for assessing animal health because they are related to stress and disease [35,36]. This means that the in ovo injection had not stressed the chicks as much. In addition, LDL cholesterol levels were significantly lower in the test lots than in the control lots. The oil extracted from BSF larvae contains compounds that can help lower LDL cholesterol levels. One such compound present in the oil, constituting 19% of its composition in this study, is linoleic acid (also known as octadecadienoic acid). In an experiment conducted by Mona et al. [37], linoleic acid was injected into the air chamber of broiler chicken eggs on the 18th day of incubation, resulting in a reduction in LDL levels. These works are consistent with those of Kisun et al. [38], who found that administering 0.5 g of linoleic acid daily to rabbits led to a decrease in blood LDL concentrations. LDL cholesterol is known as bad cholesterol, whose excess in an organism leads to cardiovascular disease. Thus, the in ovo injection of maggot oil from BSF likely reduces the risk of chickens’ cardiovascular disease by lowering the LDL cholesterol level. Apart from the positive effect of maggot oil on the health of the chicks through the biochemical blood parameters, it can also improve the quality of the chicken meat since the LDL cholesterol has been lowered [39].

## 5. Conclusions

Fats are essential nutrients for the proper functioning of the body and provide more energy than proteins or carbohydrates. In animal nutrition, edible oils are often used as an energy source. In the present study, the oil from BSF larvae was used as an energy source for in ovo injection into broiler chicken eggs. The growth performance of chicks was improved, and the blood LDL cholesterol level was decreased. The injection of 0.1 mL BSF maggot oil on the 17th day of incubation resulted in 0% embryonic mortality and 100% hatching success. BSF larvae oil is an energy source for in ovo injection, with a dose of 0.1 mL on the 17th day of incubation for the improvement of chicks’ production.

## Figures and Tables

**Table 1:** Fatty acid composition of black soldier fly maggot oil.

Fatty Acids	Quantity (%)	Fatty Acids	Quantity (%)
C12:0 Dodecanoic acid	27.7	C20:4n6 Eicosapentaenoic acid	0.508
C14:0 Etradecanoic acid	5.15	C20:5n3 Eicosapentaenoic acid	0.253
C14:1 Tetradecaenoic acid	0.0968	C22:0 Docosanoic acid	0.0224
C15:0 Pentadecanoic acid	0.156	C22:6n3 Docosahexaenoic acid	0.0379
C16:0 Hexadecanoic acid	14.8	C20:1n11t Trans-11-eicosenoic acid	0.044
C16:1 Hexadecenoic acid	2.11	C18:3n9c, 12c, 15t+ C18:3n9c, 12t, 15t Cis-9,12-trans-15+cis-9-trans 12,15-Octadecatrienoic acid	0.098
C17:0 Heptadecanoic acid	0.216	Trans fatty acids	0.14
C18:0 Octadecanoic acid	3.36	Total fatty acids	99.9
C18:1n9c Octadecanoenoic acid	22.6	Saturated fatty acids	52.6
C18:2n6c Octadecadienoic acid	19.0	Monounsaturated fatty acids	25.0
C18:3n6 γ-Octadecatrienoic acid	0.116	Polyunsaturated fatty acids	22.4
C18:3n3 α-Octadecatrienoic acid	2.34	Omega-3	2.66
C20:0 Eicosic acid	0.0779	Omega-6	19.7
C20:1 Eicosaenoic acid	0.188	Omega-9	20.8
C20:2 Eicosanoic acid	0.0515		
C20:3n6 Eicosatrienoic acid	0.0292		
C20:3n3 Eicosatrienoic acid	0.0294		

**Table 2:** Effect of in ovo injection of maggot oil from black soldier flies at the 11th day of incubation on biochemical parameters during the startup phase.

Parameters	Treatments							*p* Value
	CON−	CON+	L0.1	L0.2	L0.3	L0.4	L0.5	
ALT (UI/L)	1.40 ± 0.60 ^b^	2.00 ± 0.83 ^ab^	5.00 ± 0.44 ^a^	3.00 ± 0.44 ^ab^	2.60 ± 0.24 ^ab^	5.00 ± 0.44 ^a^	2.80 ± 0.58 ^ab^	0.0002
AST (UI/L)	250 ± 0.44 ^c^	255 ± 0.44 ^b^	284 ± 0.44 ^a^	251 ± 0.44 ^c^	242 ± 0.44 ^d^	235 ± 0.44 ^e^	232 ± 0.44 ^e^	<0.0001
TP (g/L)	31.44 ± 2.53	35.05 ± 2.82	34.31 ± 2.66	29.90 ± 1.57	27.08 ± 1.59	33.68 ± 0.92	31.74 ± 2.83	0.2068
ALB (g/L)	27.16 ± 2.41	27.52 ± 1.95	23.70 ± 1.72	23.96 ± 0.88	23.10 ± 1.13	25.36 ± 0.54	22.60 ± 0.38	0.1421
GLU (mmol/L)	10.18 ± 0.25 ^a^	10.06 ± 0.40 ^a^	6.50 ± 1.42 ^b^	11.12 ± 0.22 ^a^	11.18 ± 0.22 ^a^	12.96 ± 0.004 ^a^	9.95 ± 0.61 ^a^	<0.0001
TG (mmol/L)	1.08 ± 0.01 ^b^	1.04 ± 0.03 ^bc^	0.95 ± 0.004 ^c^	1.06 ± 0.03 ^bc^	0.93 ± 0.02 ^c^	1.46 ± 0.004 ^a^	0.89 ± 0.03 ^c^	<0.0001
TCHO (mmol/L)	4.03 ± 0.14 ^ab^	3.58 ± 0.23 ^b^	2.90 ± 0.04 ^c^	3.55 ± 0.07 ^bc^	4.28 ± 0.06 ^a^	4.06 ± 0.004 ^ab^	3.32 ± 0.25 ^bc^	<0.0001
HDL (mmol/L)	2.86 ± 0.10 ^ab^	2.46 ± 0.19 ^bc^	2.06 ± 0.004 ^c^	2.50 ± 0.02 ^bc^	3.04 ± 0.06 ^a^	2.41 ± 0.004 ^bc^	2.48 ± 0.18 ^bc^	<0.0001
LDL (mmol/L)	0.92 ± 0.03 ^b^	0.83 ± 0.05 ^b^	0.59 ± 0.004 ^c^	0.77 ± 0.05 ^b^	0.84 ± 0.03 ^b^	1.16 ± 0.004 ^a^	0.58 ± 0.05 ^c^	<0.0001

CON−: Lot control without injection and without drilling; CON+: Lot control drilled but without injection; L0.1: Lot injected with 0.1 mL of BSF maggot oil; L0.2: Lot injected with 0.2 mL of BSF maggot oil; L0.3: Lot injected with 0.3 mL of BSF maggot oil; L0.4: Lot injected with 0.4 mL of BSF maggot oil; L0.5: Lot injected with 0.5 mL of BSF maggot oil. ALT: Alanine aminotransferase, AST: Aspartate aminotransferase, TP: Total protein, ALB: Albumin, GLU: Glucose, TG: Triglyceride, TCHO: Total cholesterol, HDL: High-density lipoprotein, LDL: Low-density lipoprotein, UI/L: International unit per liter, g/L: Gram per liter, mmol/L: Millimole per liter. The results are represented as the mean value with pooled SEM (*n* = 6). Numbers with different letters in the same line indicate a significant difference (*p* < 0.05).

**Table 3:** Effect of in ovo injection of maggot oil from black soldier flies at the 14th day of incubation on biochemical parameters during the startup phase.

Parameters	Treatments							*p* Value
	CON−	CON+	L0.1	L0.2	L0.3	L0.4	L0.5	
ALT (UI/L)	4.40 ± 0.24	5.40 ± 0.6	4.40 ± 0.92	3.40 ± 0.50	4.00 ± 0.31	4.00 ± 0.89	3.00 ± 0.31	0.1710
AST (UI/L)	238 ± 0.44 ^d^	239 ± 0.44 ^d^	261 ± 0.44 ^a^	263 ± 0.44 ^a^	251 ± 0.44 ^b^	244 ± 0.44 ^c^	251 ± 0.44 ^b^	<0.0001
TP (g/L)	29.42 ± 3.46	31.26 ± 2.61	23.78 ± 2.03	29.92 ± 2.34	28.96 ± 3.22	25.74 ± 1.59	33.68 ± 1.66	0.1485
ALB (g/L)	22.64 ± 1.65	25.36 ± 1.66	20.10 ± 1.36	25.54 ± 2.05	23.70 ± 1.75	21.68 ± 1.93	26.96 ± 1.00	0.0550
GLU (mmol/L)	10.41 ± 0.53	12.04 ± 0.66	10.01 ± 0.66	10.99 ± 0.57	11.11 ± 0.51	11.23 ± 0.55	11.56 ± 0.60	0.2787
TG (mmol/L)	0.93 ± 0.02	0.92 ± 0.03	1.05 ± 0.09	0.99 ± 0.03	0.9 ± 0.04	0.91 ± 00	1.02 ± 0.05	0.2415
TCHO (mmol/L)	3.93 ± 0.27	4.30 ± 0.12	3.66 ± 0.13	3.74 ± 0.16	3.42 ± 0.35	3.55 ± 0.24	4.07 ± 0.17	0.1187
HDL (mmol/L)	2.76 ± 0.16	2.92 ± 0.09	2.52 ± 0.10	2.67 ± 0.12	2.46 ± 0.20	2.55 ± 0.16	2.77 ± 0.11	0.2947
LDL (mmol/L)	0.80 ± 0.15	1.07 ± 0.11	0.83 ± 0.11	0.80 ± 0.06	0.75 ± 0.10	0.62 ± 0.10	0.97 ± 0.05	0.1191

CON−: Lot control without injection and without drilling; CON+: Lot control drilled but without injection; L0.1: Lot injected with 0.1 mL of BSF maggot oil; L0.2: Lot injected with 0.2 mL of BSF maggot oil; L0.3: Lot injected with 0.3 mL of BSF maggot oil; L0.4: Lot injected with 0.4 mL of BSF maggot oil; L0.5: Lot injected with 0.5 mL of BSF maggot oil. ALT: Alanine aminotransferase, AST: Aspartate aminotransferase, TP: Total protein, ALB: Albumin, GLU: Glucose, TG: Triglyceride, TCHO: Total cholesterol, HDL: High-density lipoprotein, LDL: Low-density lipoprotein, UI/L: International unit per liter, g/L: Gram per liter, mmol/L: Millimole per liter. The results are represented as the mean value with pooled SEM (*n* = 6). Numbers with different letters in the same line indicate a significant difference (*p* < 0.05).

**Table 4:** Effect of in ovo injection of maggot oil from black soldier flies at the 17th day of incubation on biochemical parameters during the startup phase.

Parameters	Treatments							*p* Value
	CON−	CON+	L0.1	L0.2	L0.3	L0.4	L0.5	
ALT (UI/L)	2.80 ± 0.66	2.60 ± 0.50	3.80 ± 0.73	3.00 ± 0.54	4.20 ± 0.73	3.20 ± 0.2	2.80 ± 0.37	0.4016
AST (UI/L)	245 ± 0.44 ^b^	203 ± 0.44 ^e^	246 ± 0.44 ^b^	233 ± 0.44 ^c^	229 ± 0.44 ^d^	243 ± 0.44 ^b^	264 ± 0.44 ^a^	<0.0001
TP (g/L)	29.02 ± 1.34	28.18 ± 2.72	25.84 ± 1.05	26.42 ± 2.61	28.12 ± 2.20	26.90 ± 2.99	26.00 ± 1.60	0.9233
ALB (g/L)	24.22 ± 1.36	20.62 ± 1.47	21.54 ± 0.79	21.76 ± 1.77	22.54 ± 1.00	22.46 ± 2.39	22.40 ± 1.18	0.7711
GLU (mmol/L)	11.03 ± 0.35	10.94 ± 0.59	11.58 ± 0.36	11.54 ± 0.36	11.28 ± 0.34	11.89 ± 0.41	11.91 ± 0.36	0.5258
TG (mmol/L)	0.94 ± 0.04	0.89 ± 0.04	0.91 ± 0.05	0.95 ± 0.07	0.97 ± 0.06	0.93 ± 0.04	0.91 ± 0.02	0.9578
TCHO (mmol/L)	4.18 ± 0.19	3.70 ± 0.17	4.57 ± 0.11	3.94 ± 0.20	3.90 ± 0.41	3.76 ± 0.15	3.63 ± 0.17	0.0820
HDL (mmol/L)	2.55 ± 0.18 ^b^	2.53 ± 0.07 ^b^	3.19 ± 0.07 ^a^	2.82 ± 0.11 ^ab^	2.75 ± 0.24 ^ab^	2.62 ± 0.07 ^ab^	2.65 ± 0.08 ^ab^	0.0347
LDL (mmol/L)	0.93 ± 0.10	0.83 ± 0.09	0.95 ± 0.08	0.78 ± 0.10	0.87 ± 0.17	0.88 ± 0.09	0.72 ± 0.06	0.7502

CON−: Lot control without injection and without drilling; CON+: Lot control drilled but without injection; L0.1: Lot injected with 0.1 mL of BSF maggot oil; L0.2: Lot injected with 0.2 mL of BSF maggot oil; L0.3: Lot injected with 0.3 mL of BSF maggot oil; L0.4: Lot injected with 0.4 mL of BSF maggot oil; L0.5: Lot injected with 0.5 mL of BSF maggot oil. ALT: Alanine aminotransferase, AST: Aspartate aminotransferase, TP: Total protein, ALB: Albumin, GLU: Glucose, TG: Triglyceride, TCHO: Total cholesterol, HDL: High-density lipoprotein, LDL: Low-density lipoprotein, UI/L: International unit per liter, g/L: Gram per liter, mmol/L: Millimole per liter. The results are represented as the mean value with pooled SEM (*n* = 6). Numbers with different letters in the same line indicate a significant difference (*p* < 0.05).

**Table 5:** Effect of in ovo injection of maggot oil from black soldier flies at the 11th day of incubation on biochemical parameters during the growth phase.

Parameters	Treatments							*p* Value
	CON−	CON+	L0.1	L0.2	L0.3	L0.4	L0.5	
ALT (UI/L)	3.00 ± 0.70	2.60 ± 0.81	3.60 ± 1.16	4.00 ± 0.44	4.60 ± 0.97	2.40 ± 0.24	1.80 ± 0.58	0.1731
AST (UI/L)	238 ± 2.28	237 ± 2.72	234 ± 3.63	233 ± 3.63	231 ± 3.48	227 ± 2.83	226 ± 2.45	0.0758
TP (g/L)	33.68 ± 0.52	33.92 ± 1.05	29.10 ± 1.35	36.14 ± 1.92	33.44 ± 1.80	34.74 ± 0.39	31.02 ± 2.40	0.0527
ALB (g/L)	26.66 ± 0.18	24.64 ± 0.65	24.82 ± 1.09	27.50 ± 1.55	25.18 ± 0.76	28.24 ± 0.38	24.88 ± 1.51	0.0835
GLU (mmol/L)	10.45 ± 0.31	9.88 ± 0.27	10.23 ± 0.64	10.75 ± 0.36	9.93 ± 0.64	11.49 ± 0.11	10.02 ± 0.43	0.1550
TG (mmol/L)	0.79 ± 0.03	0.74 ± 0.01	0.85 ± 0.03	0.77 ± 0.005	0.79 ± 0.03	0.82 ± 0.02	0.79 ± 0.04	0.3589
TCHO (mmol/L)	2.83 ± 0.32	2.39 ± 0.15	2.48 ± 0.17	2.92 ± 0.27	2.72 ± 0.16	2.24 ± 0.03	2.24 ± 0.34	0.2477
HDL (mmol/L)	1.95 ± 0.18	1.67 ± 0.13	1.86 ± 0.12	1.98 ± 0.15	1.92 ± 0.07	1.81 ± 0.03	1.62 ± 0.23	0.5238
LDL (mmol/L)	0.57 ± 0.03 ^a^	0.57 ± 0.04 ^a^	0.35 ± 0.03 ^b^	0.33 ± 0.05 ^b^	0.33 ± 0.04 ^b^	0.35 ± 0.04 ^b^	0.34 ± 0.03 ^b^	0.0002

CON−: Lot control without injection and without drilling; CON+: Lot control drilled but without injection; L0.1: Lot injected with 0.1 mL of BSF maggot oil; L0.2: Lot injected with 0.2 mL of BSF maggot oil; L0.3: Lot injected with 0.3 mL of BSF maggot oil; L0.4: Lot injected with 0.4 mL of BSF maggot oil; L0.5: Lot injected with 0.5 mL of BSF maggot oil. ALT: Alanine aminotransferase, AST: Aspartate aminotransferase, TP: Total protein, ALB: Albumin, GLU: Glucose, TG: Triglyceride, TCHO: Total cholesterol, HDL: High-density lipoprotein, LDL: Low-density lipoprotein, UI/L: International unit per liter, g/L: Gram per liter, mmol/L: Millimole per liter. The results are represented as the mean value with pooled SEM (*n* = 6). Numbers with different letters in the same line indicate a significant difference (*p* < 0.05).

**Table 6:** Effect of in ovo injection of maggot oil from black soldier flies at the 14th day of incubation on biochemical parameters during the growth phase.

Parameters	Treatments							*p* Value
	CON−	CON+	L0.1	L0.2	L0.3	L0.4	L0.5	
ALT (UI/L)	3.60 ± 0.7	3.60 ± 1.03	1.60 ± 0.40	2.80 ± 0.37	2.00 ± 0.31	2.20 ± 0.48	2.20 ± 0.20	0.1501
AST (UI/L)	233 ± 2.28	235 ± 2.15	232 ± 2.97	231 ± 2.97	230 ± 2.11	227 ± 1.64	224 ± 3.56	0.0737
TP (g/L)	34.54 ± 0.77	34.80 ± 1.75	34.78 ± 1.96	30.16 ± 1.62	30.82 ± 1.99	36.92 ± 2.14	35.50 ± 1.06	0.0754
ALB (g/L)	23.74 ± 0.92	25.02 ± 1.45	25.06 ± 1.27	23.52 ± 1.26	23.88 ± 1.27	26.70 ± 0.78	26.22 ± 0.97	0.3558
GLU (mmol/L)	9.03 ± 0.24	9.94 ± 0.65	9.08 ± 0.45	9.03 ± 0.32	9.09 ± 0.61	9.76 ± 0.73	9.69 ± 0.79	0.8148
TG (mmol/L)	0.75 ± 0.03	0.85 ± 0.04	0.81 ± 0.01	0.77 ± 0.01	0.90 ± 0.06	0.84 ± 0.06	0.87 ± 0.04	0.2373
TCHO (mmol/L)	2.22 ± 0.10	2.22 ± 0.10	2.49 ± 0.19	2.41 ± 0.24	2.28 ± 0.12	2.27 ± 0.19	2.15 ± 0.18	0.8161
HDL (mmol/L)	1.51 ± 0.11	1.53 ± 0.13	2.01 ± 0.19	1.93 ± 0.18	1.79 ± 0.07	1.77 ± 0.17	1.65 ± 0.18	0.2323
LDL (mmol/L)	0.69 ± 0.07 ^a^	0.69 ± 0.06 ^a^	0.45 ± 0.02 ^b^	0.46 ± 0.05 ^b^	0.45 ± 0.05 ^b^	0.48 ± 0.02 ^b^	0.46 ± 0.04 ^b^	0.0003

CON−: Lot control without injection and without drilling; CON+: Lot control drilled but without injection; L0.1: Lot injected with 0.1 mL of BSF maggot oil; L0.2: Lot injected with 0.2 mL of BSF maggot oil; L0.3: Lot injected with 0.3 mL of BSF maggot oil; L0.4: Lot injected with 0.4 mL of BSF maggot oil; L0.5: Lot injected with 0.5 mL of BSF maggot oil. ALT: Alanine aminotransferase, AST: Aspartate aminotransferase, TP: Total protein, ALB: Albumin, GLU: Glucose, TG: Triglyceride, TCHO: Total cholesterol, HDL: High-density lipoprotein, LDL: Low-density lipoprotein, UI/L: International unit per liter, g/L: Gram per liter, mmol/L: Millimole per liter. The results are represented as the mean value with pooled SEM (*n* = 6). Numbers with different letters in the same line indicate a significant difference (*p* < 0.05).

**Table 7:** Effect of in ovo injection of maggot oil from black soldier flies at the 17th day of incubation on biochemical parameters during the growth phase.

Parameters	Treatments							*p* Value
	CON−	CON+	L0.1	L0.2	L0.3	L0.4	L0.5	
ALT (UI/L)	2.40 ± 0.50	2.60 ± 0.24	1.60 ± 0.24	1.80 ± 1.11	2.40 ± 0.50	2.20 ± 0.58	2.20 ± 0.58	0.9075
AST (UI/L)	228 ± 2.28	227 ± 2.53	224 ± 2.15	223 ± 1.98	224 ± 2.54	222 ± 3.07	222 ± 3.66	0.6689
TP (g/L)	29.76 ± 1.49	35.16 ± 0.88	29.76 ± 2.53	30.40 ± 1.09	28.88 ± 1.69	29.66 ± 0.1	31.12 ± 1.31	0.1048
ALB (g/L)	21.50 ± 1.16	24.86 ± 1.06	22.80 ± 1.61	22.60 ± 0.63	22.64 ± 0.42	24.32 ± 0.29	24.22 ± 0.82	0.1885
GLU (mmol/L)	9.84 ± 1.73	10.55 ± 0.31	10.23 ± 0.24	9.49 ± 0.44	10.39 ± 0.58	10.33 ± 0.24	10.66 ± 0.32	0.9302
TG (mmol/L)	0.79 ± 0.03	0.76 ± 0.04	0.86 ± 0.06	0.93 ± 0.02	0.96 ± 0.10	0.92 ± 0.06	0.92 ± 0.06	0.2757
TCHO (mmol/L)	2.32 ± 0.07	2.37 ± 0.19	2.47 ± 0.30	2.45 ± 0.14	2.35 ± 0.19	2.49 ± 0.21	2.50 ± 0.20	0.9914
HDL (mmol/L)	1.54 ± 0.09	1.54 ± 0.18	2.04 ± 0.27	2.06 ± 0.10	1.91 ± 0.16	2.01 ± 0.17	1.91 ± 0.22	0.2177
LDL (mmol/L)	0.78 ± 0.05 ^a^	0.76 ± 0.06 ^a^	0.42 ± 0.04 ^b^	0.45 ± 0.04 ^b^	0.46 ± 0.05 ^b^	0.48 ± 0.05 ^b^	0.48 ± 0.04 ^b^	<0.0001

CON−: Lot control without injection and without drilling; CON+: Lot control drilled but without injection; L0.1: Lot injected with 0.1 mL of BSF maggot oil; L0.2: Lot injected with 0.2 mL of BSF maggot oil; L0.3: Lot injected with 0.3 mL of BSF maggot oil; L0.4: Lot injected with 0.4 mL of BSF maggot oil; L0.5: Lot injected with 0.5 mL of BSF maggot oil. ALT: Alanine aminotransferase, AST: Aspartate aminotransferase, TP: Total protein, ALB: Albumin, GLU: Glucose, TG: Triglyceride, TCHO: Total cholesterol, HDL: High-density lipoprotein, LDL: Low-density lipoprotein, UI/L: International unit per liter, g/L: Gram per liter, mmol/L: Millimole per liter. The results are represented as the mean value with pooled SEM (*n* = 6). Numbers with different letters in the same line indicate a significant difference (*p* < 0.05).

## Data Availability

Data will be made available upon request.
